# The War and the Future of Poor-Law Infirmaries

**Published:** 1917-06-09

**Authors:** 


### THE WAR AND THE FUTURE OF POOR-LAW INFIRMARIES.

At the outset of the war a public protest was raised against the treatment of wounded soldiers in Poor-Law institutions. We fear that their numbers rather than the conscience of the Government produced a change whereby many Poor-Law infirmaries were taken over by the military, the taint of pauperism was removed, and the wounded soldier became a patient in a re-christened and re-formed military hospital. The discharged soldier, however, has always been the despised and rejected of officials, and the discharged soldier, technically capable of work, but really incapacitated, and thrown aside with a trifling pension, is once more knocking at the infirmary's doors. The natural protest has again been raised, if less loudly than before.

The real complaint of the discharged soldier is that it is to the Government's interest to class him as partially disabled because then it need only give him a few shillings a week, whereupon he is forced to apply for Poor Relief, if, as is frequently the case, he is, in fact, wholly incapacitated. This state of affairs is a disgrace to the nation and is opposed to the wishes of all classes in the United Kingdom. The Government should end it forthwith. Is there no member of Parliament patriotic enough to hammer away until this shameful state of affairs is ended? So far, however, as institutional treatment is concerned, the Rev. L. R. Phelps, who has had wide personal experience of the workings of the Poor Law as member and chairman of the Oxford Board of Guardians, makes an important proposal. "Why," he asks, "should not our Poor-Law infirmaries be purged of all taint of pauperism and become rate-supported hospitals, admission to which should be determined not, as now, by [technical] destitution, but by severity of disease?" Mr. Phelps enforced his proposal by remarking that under the Insurance Act the voluntary hospitals are hard put to it to provide all the institutional treatment required.

We welcome so well informed an advocate of a policy we proposed years ago, when Sir Henry Burdett advocated it many times in his reports on Poor-Law infirmaries, and in his speeches, frequently. Sir Henry's proposal is that after the war our Poor-Law infirmaries shall be removed from the Poor Law and become State hospitals. Such a proposal has become more probable since the war, because the war has created in the discharged soldier a class of patients which can never be subjected to "the depressing yoke of the Poor Law" without protest. It has also given the separated infirmaries a new status. They have been used as military hospitals, and are among the best of them. They have, in fact, escaped from the Poor Law through the war, and the recognition they have won will not be lost. When peace returns the ex-military hospital will be besieged as a Poor-Law infirmary, largely by discharged soldiers, and the time will have arrived for it to refuse to do that work under the old stigma. Then the opportunity will have come for it to develop into the public hospital for which Sir Henry Burdett and other pioneers have contended.

